# Differential expression of interferon responsive genes in rodent models of transmissible spongiform encephalopathy disease

**DOI:** 10.1186/1750-1326-2-5

**Published:** 2007-03-16

**Authors:** Michael J Stobart, Debra Parchaliuk, Sharon LR Simon, Jillian LeMaistre, Jozef Lazar, Richard Rubenstein, J David Knox

**Affiliations:** 1 Division of Host Genetics and Prion Diseases, Public Health Agency of Canada, Canadian Science Centre for Human and Animal Health, Winnipeg, MB R3E 3R2, Canada; 2 Department of Medical Microbiology and Infectious Diseases, Faculty of Medicine, University of Manitoba, Winnipeg, MB R3E 0W3, Canada; 3 Department of Pharmacology and Therapeutics, Faculty of Medicine, University of Manitoba, Winnipeg, MB R3E 0W3, Canada; 4 Department of Dermatology and Human Molecular Genetics Center, MCW, Milwaukee, WI 53226, USA; 5 Department of Biochemistry, SUNY Downstate Medical Center, Brooklyn, NY 11203, USA

## Abstract

**Background:**

The pathological hallmarks of transmissible spongiform encephalopathy (TSE) diseases are the deposition of a misfolded form of a host-encoded protein (PrP^res^), marked astrocytosis, microglial activation and spongiosis. The development of powerful gene based technologies has permitted increased levels of pro-inflammatory cytokines to be demonstrated. However, due to the use of assays of differing sensitivities and typically the analysis of a single model system it remained unclear whether this was a general feature of these diseases or to what extent different model systems and routes of infection influenced the relative levels of expression. Similarly, it was not clear whether the elevated levels of cytokines observed in the brain were accompanied by similar increases in other tissues that accumulate PrP^res^, such as the spleen.

**Results:**

The level of expression of the three interferon responsive genes, Eif2ak2, 2'5'-OAS, and Mx2, was measured in the brains of Syrian hamsters infected with scrapie 263K, VM mice infected with bovine spongiform encephalopathy and C57BL/6 mice infected with the scrapie strain ME7. Glial fibrillary acidic expression confirmed the occurrence of astrocytosis in all models. When infected intracranially all three models showed a similar pattern of increased expression of the interferon responsive genes at the onset of clinical symptoms. At the terminal stage of the disease the level and pattern of expression of the three genes was mostly unchanged in the mouse models. In contrast, in hamsters infected by either the intracranial or intraperitoneal routes, both the level of expression and the expression of the three genes relative to one another was altered. Increased interferon responsive gene expression was not observed in a transgenic mouse model of Alzheimer's disease or the spleens of C57BL/6 mice infected with ME7. Concurrent increases in TNFα, TNFR1, Fas/ApoI receptor, and caspase 8 expression in ME7 infected C57BL/6 mice were observed.

**Conclusion:**

The identification of increased interferon responsive gene expression in the brains of three rodent models of TSE disease at two different stages of disease progression suggest that this may be a general feature of the disease in rodents. In addition, it was determined that the increased interferon responsive gene expression was confined to the CNS and that the TSE model system and the route of infection influenced the pattern and extent of the increased expression. The concurrent increase in initiators of Eif2ak2 mediated apoptotic pathways in C57BL/6 mice infected with ME7 suggested one mechanism by which increased interferon responsive gene expression may enhance disease progression.

## Background

Transmissible spongiform encephalopathies (TSEs) are slowly progressive, invariably fatal degenerative disorders of the central nervous system. The hallmark of these diseases is the accumulation of misfolded isoforms of a host-encoded protein, PrP or prion protein. The disease related isoforms, PrP^res^, are derived from the host PrP^c ^protein by a post-translational process and can be distinguished from the endogenous protein by their partial resistance to digestion by proteinase K [1,2].

TSEs share similarities with a 'family' of neurodegenerative diseases, including Alzheimer's, that are characterized by the deposition of insoluble amyloid plaques composed of host encoded proteins, astrocytosis and microglial activation [3-5]. TSE diseases are distinguished by the fact that in addition to familial and spontaneous forms of the disease they can be transmitted iatrogenically or through dietary exposure to contaminated tissue [6]. Another feature specific to TSEs is the existence of multiple strains, each having a distinct incubation period, pattern of PrP^res ^deposition and vacuolation profile in a given host [7-10].

Studies examining TSE disease induced gene expression have demonstrated an increased expression of markers for astrocyte and microglial activation and proliferation, as well as pro-inflammatory cytokines [11-17]. Collectively, these studies are in part responsible for the view that TSE diseases evoke an atypical inflammatory response. Unfortunately, due to the use of assays of differing sensitivities and typically the analysis of a single model system it is unclear to what extent different model systems and routes of infection influence the relative levels of expression. In addition, it is unknown whether there is a coincident increase in expression of some of the same genes in peripheral tissues, such as the spleen, where significant amounts of PrP^res ^are observed [18]. To explore these issues we chose to examine the expression of two interferon responsive genes, 2'5'-oligoadenylate synthetase (2'5'-OAS) and myxovirus resistance gene 2 (Mx2) whose increased expression had previously been described in one or more models of TSE disease [19,20]. The expression of an additional interferon responsive gene not previously analyzed and key mediator of apoptotic pathways, eukaryotic translation initiation factor 2 alpha kinase 2 (Eif2ak2), was also determined [21].

In this study, we take advantage of the fact that each TSE strain, once adapted to a particular host, follows a very predictable disease course [22-26]. The expression levels of 2'5'-OAS, Mx2 and Eif2ak2 genes were measured at the time of the onset of symptoms as well as at the terminal stage of disease in four TSE models as well as a transgenic model of Alzheimer's disease by quantitative real-time PCR (qPCR). qPCR is a highly sensitive method of determining the cycle numbers at which exponential amplification occurs and the amplification of a single specified product can be confirmed by gel electrophoresis. Unlike conventional PCR and Northern blot analysis, the qPCR results, based solely upon the cycles during which exponential amplification occurs, enable the relative or absolute template copy numbers to be determined from a standard curve [27-29]. This permits a direct comparative measurement of the influence of TSE model system, route of infection, and tissue specific effects on the level of expression of these three genes, even if the differences are minute.

## Results

The increased expression of the interferon (IFN) responsive genes, 2'5'-OAS, and Mx proteins previously reported in mouse models suggested that the induction of interferon responsive genes may be a general response to TSE infection [19,20]. To investigate this possibility we compared the whole brain gene expression profiles observed in Syrian hamsters infected with scrapie 263K, VM mice infected with bovine spongiform encephalopathy (BSE) and C57BL/6 mice infected with the scrapie strain ME7. In order to check the expression of Eif2ak2 in all three models a fragment of the hamster Eif2ak2 mRNA was amplified and cloned, using primers based on homologous sequences found in the human, mouse and rat.

**Figure 1:**
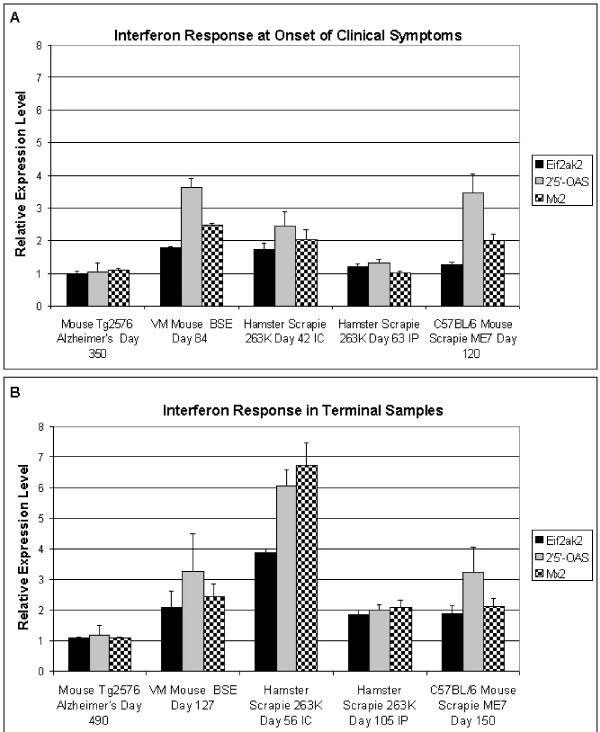
**Expression levels of interferon responsive genes in four different models of amyloid disease**. Pooled samples of total RNA isolated from the whole brains of diseased or age-matched control animals were used to determine relative gene expression levels by quantitative real-time PCR. The histograms represent the average of the means of three independent quantitative real-time PCR reactions, done in duplicate, for each gene ± standard deviation. All plotted data represent gene expression levels in diseased animals relative to gene expression levels in age and strain-matched control animals. The days post infection (dpi) indicated correspond to the times at which the onset of clinical symptoms and terminal stage disease are observed in the different model systems. At the onset of clinical symptoms (panel A) significantly higher expression levels of the three interferon responsive genes were observed in all the models of TSE disease via the IC route (p ≤ 0.008). No significant increase was observed in the hamster scrapie 263K 63 day IP sample or the similar disease stage transgenic mouse model of Alzheimer's disease. At the terminal stage of disease (panel B) the expression of the three interferon responsive genes was significantly increased in all rodent models of TSE disease (p ≤ 0.002) but not in the Alzheimer's disease model (p ≤ 0.32).

In the three model systems infected via the IC route a statistically significant (p < 0.008) increase in the expression of all three IFN responsive genes was observed at the onset of clinical disease (Figure 1A). In addition, the pattern of increased expression was similar in the three IC infected rodent models. The greatest increase in expression was exhibited by 2'5'-OAS followed by Mx2 and Eif2ak2. No statistically significant increase in the expression of the three interferon responsive genes was observed in either the hamster IP model or the transgenic mouse model of Alzheimer's disease at the onset of clinical symptoms.

At the terminal stage of the disease a statistically significant (p ≤ 0.002) increased expression of the three IFN responsive genes was observed in all three rodent models of TSE disease and by both routes of infection (Figure 1B). In the mouse models of TSE disease the levels of 2'5'-OAS and Mx2 expression did not increase further as the mice progressed from the onset of clinical signs to the terminal stage of the disease. Similarly, no further increase in Eif2ak2 expression was observed in the mouse model of BSE, but a statistically significant 67% increase in Eif2ak2 expression (p = 0.00052) was observed in the scrapie mouse model. The pattern of increased expression of the interferon responsive genes relative to one another in these two models remained the same as that observed at the onset of clinical signs. In contrast, in the IC hamster model all three genes increased more than twofold as the animals progressed on to the terminal stage of disease and therefore to a significantly higher level than that observed in the mouse models. The more than three fold increase in Mx2 expression over this period also caused the pattern of increased interferon responsive gene expression in the IC hamster model to change from that observed at the onset of disease (Figure 1). In terminal stage hamsters infected with the scrapie strain 263K, Mx2 exhibited the greatest increase in expression followed by 2'5'-OAS and Eif2ak2 respectively. The pattern of interferon responsive gene expression displayed by the IC infected hamsters at the terminal stage of the disease was shared by terminal stage hamsters infected with the same scrapie strain via the IP route. The absolute levels of increased expression in the IP model were less than that observed when administered via the IC route. No significant change in the expression of the three IFN responsive genes was observed in this transgenic mouse model of Alzheimer's disease at the terminal stage of the disease when compared to age matched isogenic controls (Figure 1B).

**Figure 2:**
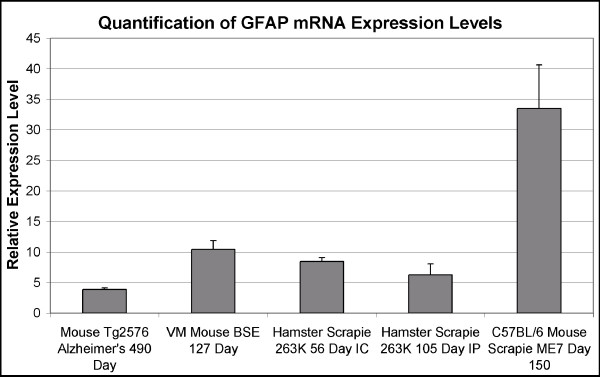
**Expression level of GFAP in four different models of amyloid disease**. Pooled samples of total RNA isolated from the whole brains of diseased or age-matched control animals were used to determine relative GFAP expression levels by quantitative real-time PCR. The histograms represent the average of the means of three independent quantitative real-time PCR reactions, done in duplicate, ± standard deviation. All plotted data represent expression levels in diseased animals relative to gene expression levels in age and strain-matched control animals. A statistically significant increase in GFAP expression was observed in all terminal disease models (p = 3.44E^-5 ^for Alzheimer's model, p = 0.000364 for VM BSE mice, p = 3.24E^-5 ^for Hamster 263K 56 IC, p = 0.0068 for Hamster 263K IP, and p = 0.0014 for C57BL/6 ME7 mice).

To test the specificity of the interferon responsive gene up regulation in the models of TSE disease and not the mouse model of Alzheimer's disease the level of glial fibrillary acidic protein (GFAP) mRNA was determined (Figure 2). The four fold or more increase in GFAP expression observed in all models is consistent with the well established astrocytosis known to occur in the mouse model of Alzheimer's disease as well as the models of TSE disease [30].

**Figure 3:**
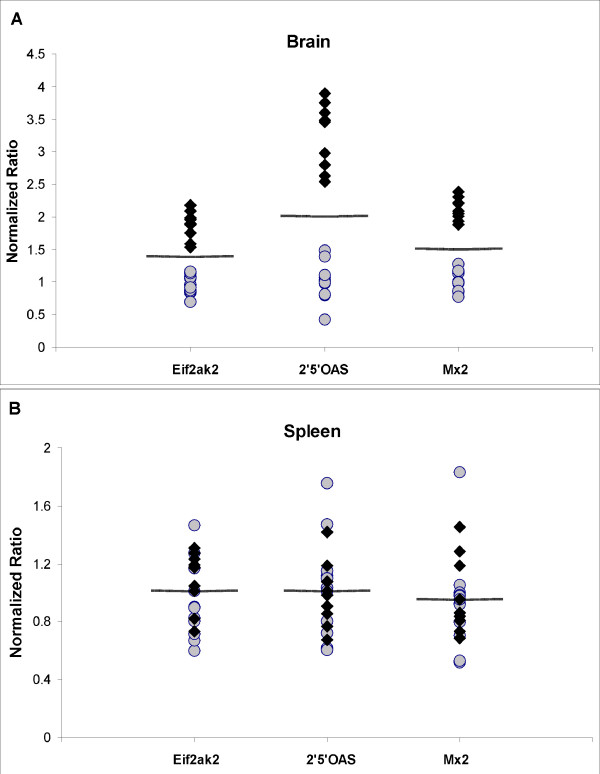
**IFN responsive gene expression in the brains and spleens of individual ME7 infected and mock infected C57BL/6 mice**. cDNA from whole brains and spleens of 9 ME7 infected and 10 mock infected C57BL/6 mice that were analyzed in a blinded fashion. Equal amounts of cDNA from the 10 mock infected samples were pooled to make an eleventh sample. Each data point represents the mean of three independent quantitative real-time PCR reactions for each gene normalized to the average level of expression exhibited by the mock infected samples. Filled diamonds represent those samples obtained from brains of ME7 infected C57BL/6 mice. The shaded circles represent cDNA samples obtained from age-matched mock infected C57BL/6 mice. The lines represent the mean value of the 20 samples for each gene. A clear segregation of the infected and mock-infected samples based upon the relative expression of any of the three interferon responsive genes was observed in brain tissue (A, Eif2ak2 p = 1.39E^-23^, 2'5'-OAS p = 7.04E^-21^, Mx2 p = 7.08E^-26^). A random distribution of infected and mock infected samples derived from the corresponding spleen tissue relative to the mean of the sample set was observed (B, Eif2ak2 p = 0.75, 2'5'-OAS p = 0.80, Mx2 p = 0.48).

The above results were generated using pooled total RNA derived from whole brain samples. In order to determine how consistently the increased expression of the IFN responsive genes would be observed in individual mice, a panel of 9 terminal stage ME7 infected C57BL/6 mice and 10 mock-infected age-matched controls were analyzed. An additional sample consisting of a pool of the 10 control samples was also used. In Figure 3A it can be seen that using the mean value of all the samples as the delineation mark resulted in two distinct groups, one consisting of 9 samples and the other consisting of 11 samples. All the samples that fell above the mean value represented infected samples and those below represented mock infected control samples. A two-way two-sample T-test assuming equal variance was used to determine the degree of significant difference between the infected and control groups for each of the three IFN responsive genes. The calculated p-values for these groupings were p = 1.39E^-23 ^for Eif2ak2, p = 7.04E^-21 ^for 2'5'-OAS and p = 7.08E^-26 ^for Mx2 as calculated using the Data Analysis package in Microsoft Excel^©^. Therefore, with a high degree of confidence, individual infected samples at the terminal stage of disease can be distinguished from uninfected samples based upon qPCR expression analysis of Eif2ak2, 2'5'-OAS, and Mx2.

In order to determine if a change in the interferon responsive gene expression levels could be seen in a peripheral tissue known to accumulate high levels of PrP^res^, total RNA isolated from the spleens of the same 9 ME7 infected C57BL/6 mice and 10 mock infected C57BL/6 controls were used to create a second panel. These samples were analyzed in the same manner as the brain samples and this result demonstrated that the levels of mRNA for Eif2ak2, 2'5'-OAS and Mx2 in the spleen were not significantly affected by TSE disease (Figure 3B).

**Figure 4:**
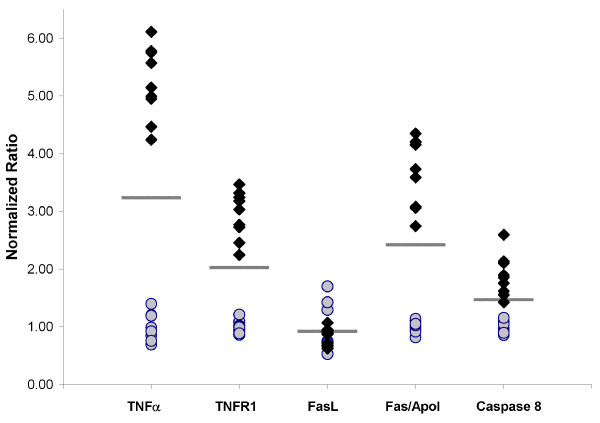
**Increase pro-apoptotic gene expression in the brains of individual ME7 infected and mock infected C57BL/6 mice**. cDNA from whole brains of 9 ME7 infected and 8 mock infected C57BL/6 mice were analyzed in a blinded fashion. Each data point represents the mean of three independent quantitative real-time PCR reactions for each gene normalized to the average level of expression exhibited by the mock infected samples. Filled diamonds represent those samples obtained from brains of ME7 infected C57BL/6 mice. The shaded circles represent cDNA samples obtained from age-matched mock infected C57BL/6 mice. The lines represent the mean value of the 17 samples for each gene. A clear segregation of the infected and mock-infected samples based upon the relative expression of TNFα, TNFR1, Fas/ApoI and caspase 8 was observed in brain tissue. In contrast, FasL was not significantly differentially expressed between infected and mock infected samples (TNFα p = 3.74E^-32^, TNFR1 p = 1.03E^-25^, FasL p = 0.071, Fas/ApoI p = 6.62E^-27^, and caspase 8 p = 1.37E^-15^).

In addition to their antiviral roles, both Eif2ak2 and 2'5'-OAS are known mediators of stress induced apoptosis [31,32], the major type of cell death associated with TSE-induced neurodegeneration. Eif2ak2 in particular has been implicated in the induction of the pro-apoptotic factors Fas/ApoI receptor and TNFR1 as well as their respective ligands FasL and TNFα [33,34] potentially through the Eif2ak2 mediated activation of NF-κB [35]. In addition, it has been reported that Eif2ak2 is able to trigger apoptosis through the FADD mediated activation of caspase 8 in a manner independent of Fas/ApoI and TNFR1 receptors [36]. To determine the most likely downstream signalling effects of Eif2ak2 influencing disease progression the levels of expression of these pro-apoptotic genes was measured in the panel of 9 terminal stage ME7 infected C57BL/6 mice and 8 mock-infected age-matched controls. In Figure 4 it can be observed that there is a clear and consistent induction of TNFα, TNFR1, Fas/ApoI receptor and caspase 8 in the panel of infected mice. In contrast, no significant induction of FasL was observed. The calculated p-values for these groupings were p = 3.74E^-32 ^for TNFα, p = 1.03E^-25 ^for TNFR1, p = 0.07 for FasL, p = 6.62E^-27 ^for Fas/ApoI receptor and p = 1.37E^-15 ^for caspase 8 as calculated using the Data Analysis package in Microsoft Excel^©^.

## Discussion

To the best of our knowledge, this is the first comparative study based upon quantitative real-time PCR analysis measuring the influence of model system, route of infection, and tissue on TSE induced expression of Eif2ak2, 2'5'-OAS, and Mx2 mRNA. Transgenic mice that develop Alzheimer's disease like pathology were included in the study to determine whether changes in the levels of expression of these interferon responsive genes were a general feature of amyloid diseases of the central nervous system. TSE disease and Alzheimer's disease are both slowly progressive diseases where the formation of amyloid deposits is accompanied by an inflammatory response, and extensive gliosis [37-39]. The three rodent models of TSE disease and the Tg2576 mouse model of Alzheimer's disease used in this study have all previously been shown to exhibit these characteristics, however, significant neurodegeneration is restricted to the models of TSE disease [37,40-44]. Despite the gliosis demonstrated in all the rodent disease models, no statistically significant increase in expression of the three IFN responsive genes was observed in the mouse model of Alzheimer's disease (Figure 1 and Figure 2). This indicated that increased expression of the three IFN responsive genes in brain tissue was not a general feature of these amyloid diseases.

TSE disease transmission, incubation period and disease phenotype are regulated by complicated interactions between the host genes and the specific TSE strain, however, when a TSE agent is serially passaged within a given host these disease characteristics are very stable. Using well characterized host adapted strains of TSE disease permitted tissue samples to be collected at similar stages of disease progression [22-26] leaving disease phenotype as the principle factor determining gene expression. Despite different neuropathological features, based on the pattern and degree of increased expression of the three interferon responsive genes used in this study no difference was observed between the three IC infected models at the onset of clinical symptoms (Figure 1A). Only at the terminal stage of the disease did the magnitude of the increase and the relative amounts of the three genes to one another distinguish the IC infected hamster model from the two mouse models that remained remarkably similar to one another (Figure 1B).

In the hamster model the route of infection played a dominant role in the magnitude of the increase in expression observed. Following peripheral infection no statistically significant increase in interferon responsive gene expression was observed until the terminal stage of the disease. Though the increase observed in the terminal stage IP infected hamsters was less robust than that exhibited by the IC infected animals the pattern of expression remained the same and suggested that the interaction of TSE strain and host dependent factors, rather than the route of infection, governed the pattern of expression of the three interferon responsive genes analyzed (Figure 1B). The increased expression of the interferon responsive genes following IP infection also indicated that their induction was a result of the disease rather than the IC infection procedure and the associated T lymphocytes observed up to 12 weeks post inoculations at the IC injection site[39].

The 2.0–6.5 fold increase in Mx2 and 2'5'-OAS detected by qPCR is relatively modest, but comparable to values reported previously [19,20]. In contrast, microarray profiling of scrapie infected brain tissue has not identified the three IFN responsive genes reported here as differentially expressed though increased expression of other interferon responsive genes was observed [45,46]. The method probably had more influence than TSE strain differences on the discrepancies observed as one of these studies included C57BL/6 mice infected I.C. with the scrapie strain ME7. In addition, the prior detection of increased expression of Eif2ak2 in TSE infected brain tissue by conventional PCR required the isolation of activated microglia [19]. Both instances attest to the sensitivity of the qPCR method. The ability to detect small differences in mRNA expression in whole brain also avoids the potential introduction of sample bias during the isolation of a particular cell type or brain region.

To demonstrate that the differential expression observed was not due to a subset of samples within the pooled RNA a set of 10 biological replicates were analyzed. The results obtained from individual ME7 infected brain samples confirmed that they all clearly exhibited increased expression of the three interferon responsive genes relative to mock infected controls (Figure 3A). In addition each biological replicate exhibited the same pattern of expression as was observed with the pooled mRNA derived from ME7 infected samples. The mRNA with the highest relative increase in expression was 2'5'-OAS followed by Mx2 and Eif2ak2 respectively. This set of experiments further supported the suggestion that TSE induced induction of these interferon responsive genes in brain tissue is a general feature of the disease in rodents.

Although the principal effects of the disease are limited to the CNS, there is considerable deposition of the misfolded PrP^c ^isoform, PrP^res^, in peripheral lymphoreticular tissue and in particular the spleen [47,48]. The suggestion that it is the microglial cells of the brain that are primarily responsible for the expression of Eif2ak2 and 2'5'-OAS made us question whether the cells of macrophage lineage present in the spleen would respond similarly to infective PrP^res ^deposition. To answer this question the spleens of the biological replicates described above were tested for expression levels of these three interferon responsive genes. No differential expression of Eif2ak2, 2'5'-OAS or Mx2 was observed following qPCR analysis of mRNA isolated from the spleens (Figure 3B). Despite the sensitivity of qPCR demonstrated in the analyses of the brain, we cannot exclude the possibility that these genes are induced in some minor spleen cell population. Nonetheless, the results obtained suggest that induction of the three interferon responsive genes is not a generalized response to PrP^res ^deposition, but instead appears to be confined to the primary site of the disease, the CNS.

In a disease where the infectious agent is widely accepted to be an aberrantly folded isoform of the host encoded protein, PrP^c ^[49], the consequence of the induction of potent anti-viral factors on disease progression is unknown. Our results suggest that one consequences of increased Eif2ak2 expression is a positive effect on disease progression through the activation of the stress induced TNFα/TNFR1 apoptotic pathway. The abundance of FasL is regulated by post translation mechanisms [50,51] that would go unnoticed in our screen of mRNA levels. Therefore, the increased expression of Fas/Apo1 could be accompanied by increased FasL abundance resulting in the activation of both external apoptotic pathways.

Conclusion

The results suggested that the induction of the interferon responsive genes, Eif2ak2, 2'5'-OAS, and Mx2, may be a general feature of rodent models of TSE disease that may contribute to disease progression through the activation of stress induced apoptotic pathways. Based upon the TSE models analyzed the route of infection primarily influenced the extent of the increased expression observed while strain and host specific factors determined the pattern of expression

The absence of an induction of the interferon responsive genes in either the Alzheimer's disease model or the spleens of infected animals indicated that amyloid deposition in the brain or an accumulation of PrP^res ^in the lymphoreticular system are not sufficient to elicit this response. Instead the induction of the interferon responsive genes must result from an interaction of the infectious agent with as yet unidentified host specific factors in the CNS.

## Methods

### Experimental animals

An internal review of all animal experiments was done prior to the commencement of the work to ensure that the procedures were carried out in accordance with the guidelines of the Canadian Council on Animal Care.

Inoculated infective material for all experiments consisted of rodent-adapted TSE strains corresponding to the appropriate rodent model. Isolation of RNA from animals at a specific age were based upon previously determined age of disease onset studies [22-26].

RNA samples extracted from the brains of scrapie 263K infected and mock infected Syrian hamsters 42 and 56 days following intracerebral (IC) inoculation, scrapie 263K infected and mock infected Syrian hamsters aged 63 and 105 days following intraperitoneal (IP) inoculation, and BSE infected and mock infected VM mice 84 and 127 days post IC inoculation were graciously provided by the laboratory of Dr. Robert Rohwer (VAMC Baltimore, MD). A total of 30 RNA samples from each group at each time point were pooled together to control for biological variation. The RNA of brains and spleens of scrapie ME7 infected and mock infected C57BL/6 mice aged 120 (onset of symptoms) and 150 (terminal stage disease) days post infection were prepared at the CSCHAH. The pooled samples consisted of RNA isolated from 10 mice. RNA samples from the brains of four Tg2576 transgenic Alzheimer mice and isogenic controls (strain C57BL/6-SJL) aged 350 days or from the brains of three 490 day old mice were used as a pooled sample (Taconic) [52]. All RNA was extracted from whole brains using RNeasy^® ^Lipid Tissue Midi Kit from Qiagen and reverse transcribed to cDNA as described below.

C57BL/6 mice 6–8 weeks old were anaesthetized with isoflurane prior to being IC infected with 20 μL, 1% w/v brain homogenate in phosphate buffered saline (PBS). The brain homogenate used for scrapie infection was prepared from terminal stage C57BL/6 mice that had been previously IC infected with strain ME7 (TSE Resource Centre, Newbury, U.K.). Age matched controls were inoculated with uninfected brain homogenate. Prior to injection the homogenates were prepared by sonicating three times for 30 seconds at amplitude setting 40 (ColeParmer Ultrasonic Processor, model CP-130), then centrifuged at 500 × g for 10 minutes.

Infected mice were observed for clinical signs throughout the course of the disease. At the days indicated infected mice and the corresponding mock infected mice serving as controls were euthanized by cervical dislocation and the brain and spleen tissue samples collected. RNA was extracted from the brain and spleen tissues using Qiagen RNeasy Lipid Tissue Midi kit according to manufacturers specifications. Two micrograms of total RNA from each sample was reverse transcribed as described below. Reactions were then purified using Microcon 30 columns (Millipore Corp. Cat. #42410) according to the manufacturers instructions. The resultant cDNA samples were quantified on a NanoDrop ND-1000 (NanoDrop Technologies) and the concentrations adjusted to 25 ng μl^-1^.

### Generation of cDNA template from total RNA samples for calibrator construction

Two micrograms of total RNA from each sample was reverse transcribed for 1 hour at 42°C in a 20 μL reaction containing 50 mM Tris pH 8.3, 75 mM KCl, 3 mM MgCl_2_, 10 mM DTT, 0.5 μg oligo dT primer, 0.5 mM each dNTP, 40U RNaseOUT RNase inhibitor, and 200 U SuperScript III RNase H^- ^reverse transcriptase (Invitrogen Cat.#18080-044). Reactions were inactivated by heating at 70°C for 15 minutes. RNA complimentary to the cDNA was removed by the addition of 2 U of *E. coli *RNase H (Invitrogen Cat.#18021-071) and incubation at 37°C for 20 minutes. Reactions were then purified using a Qiagen PCR purification kit according to manufacturers instructions. All cDNA samples were adjusted to 25 ng μL^-1^.

### Cloning of hamster Eif2ak2 for sequence determination

The sequence of the hamster Eif2ak2 gene was not available in the GenBank database, requiring us to amplify, clone and sequence a fragment of this gene. Regions of complete sequence homology between mouse, rat and human Eif2ak2 sequences (GenBank accession numbers BC016422, NM_019335, and NM_002759 respectively) were used to design the primer pair below to amplify an 834 base pair portion of the hamster Eif2ak2 gene.

Eif2ak2 forward: 5'-AGGTTTACATTTCAAGTT-3'

Eif2ak2 reverse: 5'-CTTTATCACAGAATTCC-3'

Using cDNA derived from Syrian hamster adult brain tissue (reverse transcription as described above, RNA isolated using the RNeasy^® ^Lipid Tissue Midi Kit from Qiagen), the fragment of interest was amplified using the following protocol. Initial denaturation at 95°C for 5 mins, followed by 35 cycles at 95°C for 45 sec, annealing at 50°C for 45 sec, extension at 72°C for 1 min, followed by a final extension at 72°C for 10 minutes in a 100 μL reaction containing 50 ng of each primer and 1.5 mM MgCl_2_. The resulting fragment was visualized on a 1% agarose gel, and then subsequently cloned into pCR^® ^2.1 vector using a TA cloning kit (Invitrogen Cat.#45-0046). The 834 base pair hamster fragment was sequenced and further analyzed using the NCBI GenBank database. It was found to have homology to Eif2ak2 in mouse, rat and human. The entire Syrian hamster Eif2ak2 sequence was obtained using the Invitrogen GeneRacer™ Kit L1500-01, and is available at GenBank: DQ645944. Primers to be used in quantitative real-time PCR analysis were chosen on the basis of this sequence (Table 1).

**Table 1:** PCR conditions for quantitative real-time PCR analysis of hamster and mouse models of TSE disease.

**Hamster**								
**Gene**	**Primer Pairs**	**Size (bp) **	**Denature 95°C (sec)**	**Anneal (°C)**	**Anneal Time (sec)**	**Extension 72°C (sec)**	**Mg2+ (mM)**	**Number Cycles**

**Mx2**	5'-TGGCGGTAGGCATTCAGG-3' 5'-TGCCAGGACCAAGTTTACAGG-3'	161	5	54	5	7	4	40
**2'5'-OAS**	5'-AGCCGCTGCCCCCACTGTA-3' 5'-TCCGAGACTGCCCTGAAGC-3'	109	5	57	5	5	3	50
**Eif2ak2**	5'-TAGGCCTTGTCAACAGTTATGCTCA-3' 5'-GCTGCTTTGCCTCCTGCTTGGTAG-3'	162	5	58	5	7	3	45
**GAPdH**	5'-ATGGCAAGTTCAAAGGCACAGTCA-3' 5'-TGGGGGCATCAGCAGAAGG-3'	231	5	60	5	11	3	40
**GFAP**	5'-GGACATCGAGATTGCCACCTAT-3' 5'-CATCCCTCATCTCCACTGTCTTTA-3'	176	5	60	5	8	3	45

**Mouse**								

**Gene**	**Primer Pairs**	**Size (bp) **	**Denature 95°C (sec)**	**Anneal (°C)**	**Anneal Time (sec)**	**Extension 72°C (sec)**	**Mg2+ (mM)**	**Number Cycles**

**Mx2**	5'-CCTGCCTGCCATCGCTGTC-3' 5'-GCCTCTCCACTCCTCTCCCTCATT-3'	160	5	60	5	11	3	40
**2'5'-OAS**	5'-GAGGCGGTTGGCTGAAGAGG-3' 5'-GAGGAAGGCTGGCTGTGATTGG-3'	312	5	60	5	11	3	40
**Eif2ak2**	5'-GTACAAGCGCTGGCAGAACTCAAT-3' 5'-AAGAGGCACCGGGTTTTGTAT-3'	124	5	60	5	11	4	40
**GAPdH**	5'-CACGGCAAATTCAACGGCACAGT-3' 5'-TGGGGGCATCGGCAGAAGG-3'	232	5	60	5	11	3	40
**GFAP**	5'-GAGCGAGCGTGCAGAGATGATGG-3' 5'-CTCCCGAAGCTCCGCCTGGTAGA-3'	166	5	60	5	11	3	45
**Caspase 8**	5'-TCTGCTGGGAATGGCTACGGTGAA-3' 5'-GTGTGAAGGTGGGCTGTGGCATCT-3'	212	5	60	5	11	3	45
**TNFα**	5'-TGCTCTGTGAAGGGAATGGGTGTT-3' 5'-AGTCCTTGATGGTGGTGCATGAGA-3'	241	5	60	5	11	3	45
**TNFα Receptor**	5'-AACCAGTTCCAACGCTACCTGAGT-3' 5'-AAGGGACGCACTCACTTTCTCTCA-3'	157	5	60	5	8	3	45
**Fas Ligand**	5'-ATCCCTCTGGAATGGGAAGACACA-3' 5'-ACCCAGTTTCGTTGATCACAAGGC-3'	91	5	63	5	6	3	45
**Fas/ApoI Receptor**	5'-TCGCCTATGGTTGTTGACCATCCT-3' 5'-TGGTATGGTTTCACGACTGGAGGT-3'	136	5	57	5	7	3	45

Conditions outlined above were used to determine the relative expression levels of the genes Eif2ak2, 2'5'-OAS, Mx2, GFAP, TNFα, TNFR1, FasL, Fas/ApoI, and caspase 8, as well as the reference gene GAPdH using the Roche LightCycler^® ^1.5.

### Construction of calibrator plasmids and standard curves for quantitative real-time PCR gene expression analysis

Plasmids containing each gene of interest were constructed and used as standards/calibrators in qPCR analysis. Amplicons representing Mx2, 2'5'-OAS, GAPdH, GFAP, Caspase 8, TNFα, TNFR1, FasL, and Fas/ApoI receptor were generated using the primers listed in Table 1 on an MJ Research thermocycler. The mouse Eif2ak2 amplicon was amplified using the forward primer 5'-GTA CAA GCG CTG GCA GAA CTC AAT-3' and the reverse primer 5'-AAG AGG CAC CGG GTT TTG TAT-3'. cDNA was generated from mouse and hamster RNA as described above to serve as template in all PCR reactions. Reaction conditions for PCR reactions were an initial denaturation at 95°C for 5 minutes, then 35 cycles of 95°C for 30 seconds, 55°C for 30 seconds, and 72°C for 45 seconds, followed by 72°C for 10 minutes. All PCR reactions contained 50 ng of forward and reverse primers, 15 mM dNTPs, 1× PCR buffer (10 mM Tris-HCL, 50 mM KCl, pH8.3), and 1 U of Taq DNA Polymerase (Roche Cat. #1 647 687). The amplicons were gel purified using QIAquick Gel Extraction kit (Qiagen) according to manufacturers specifications, then cloned into the pCR^® ^2.1 vector using a TA cloning kit (Invitrogen). Each construct was checked by restriction enzyme digestion with 20 U of EcoRI in 1× React3 buffer for 1 hour at 37°C (Invitrogen) and restriction product pattern visualized on a 1% agarose gel. All constructs were subsequently sequenced and confirmed to contain the correct gene fragment.

For generation of standard curves, plasmids were diluted to 20 pg μL^-1 ^in 10 mM Tris-HCl pH8.5. To each dilution 20 ng μL^-1 ^pGEM-NZ empty vector was added to stabilize the target plasmid concentration over time. The samples were subsequently serially diluted and amplified on the Roche LightCycler^® ^Real-Time PCR machine according to conditions in Table 1. Using the Roche LightCycler^® ^1.5 and the Relative Quantification Software version 1.0 (Roche Diagnostics), coefficient files were created from exported standard curves of target genes Mx2, 2'5'-OAS, Eif2ak2, GFAP, TNFα, TNFR1, FasL, Fas/ApoI receptor, caspase 8 and the reference gene, GAPdH. The inclusion of one dilution of the plasmid used to generate the corresponding coefficient file in each run (calibrator) controlled for run-to-run variation. The calibrator adjusted coefficient files, used to analyze all qPCR results, represent the standard curves from which the original template copy number in each sample was calculated, permitting run-to-run comparisons. The use of coefficient files also enabled normalization to GAPdH expression, taking into account any variation in the amount of cDNA added to reaction tubes.

### Real time quantification of interferon responsive genes in scrapie 263K infected Syrian hamsters, BSE infected VM mice, scrapie ME7 infected C57BL/6 mice and transgenic Alzheimer mice

Relative quantification of all cDNAs was performed using the LightCycler^® ^Fast Start DNA Master SYBR green kit, on a LightCycler^® ^1.5 Real-time PCR instrument (Roche Diagnostics). Amplification conditions utilized for qPCR of target cDNA are listed in Table 1. Subsequent to amplification, product purity was confirmed by melt curve analysis and visualization on a 1% agarose gel for fragment size. Relative expression levels were calculated using the Relative Quantification software from Roche Diagnostics, with interferon responsive gene expression levels normalized relative to GAPdH expression levels. The average fold increased expression shown in Figure 1 represents the averages of duplicate reactions from three independent trials (i.e. triplicates of duplicates). Interferon responsive gene expression levels in control samples represent a baseline expression of one, with infected sample gene expression compared to this baseline.

A two-sample two-tail t-test assuming equal variance was used to determine if a significant difference in gene expression existed between the control and infected/transgenic cDNA samples using the Data Analysis package in Microsoft Excel^©^.

### Analysis of individual ME7 infected and mock-infected C57BL/6 mouse samples

Triplicate of duplicate real time qPCR reactions were performed as described above (primer sequences and reaction conditions used are listed in Table 1). The relative expression levels (normalized to GAPdH) of the Mx2, 2'5'-OAS, Eif2ak2, GFAP, TNFα, TNFR1, FasL, Fas/ApoI receptor, caspase 8 genes were obtained using Relative Quantification Software (Roche Diagnostics). The expression level relative to the control average for each individual run was determined, and the three runs were averaged. A two-sample two-tail t-test assuming equal variance was used to determine if a significant difference in gene expression existed between the control and infected cDNA samples using the Data Analysis package in Microsoft Excel^©^.

## Competing interests

The author(s) declare that they have no competing interests.

## Authors' contributions

MJS generated the cDNA used for all qPCR results of brain-derived mRNA, performed all qPCR analysis on brain cDNA, generated all the calibrator plasmids and curves, performed the statistical analysis, sequenced the hamster Eif2ak2 mRNA, and helped draft the manuscript. DP homogenized brain and spleen tissue, and blinded the C57BL/6 mouse brain cDNA panel. SS aided in analysis and interpretation of data from the LightCycler and helped draft the manuscript. JLeM generated cDNA and performed qPCR analysis on cDNA derived from C57BL/6 mouse spleens. JL provided mRNA from control and infected VM mice and Syrian hamsters. RR provided mRNA from transgenic mouse models of Alzheimer's disease. JDK conceived of the study and its design, and primarily drafted the manuscript. All authors read and approved the final manuscript.
